# Comment on Akil et al. Nonintubated versus Intubated Lung Volume Reduction Surgery in Patients with End-Stage Lung Emphysema and Hypercapnia. *J. Clin. Med.* 2023, *12*, 3750

**DOI:** 10.3390/jcm14093062

**Published:** 2025-04-29

**Authors:** Franziska C. Trudzinski, Hans Hoffmann, Frank Langer, Konstantina Kontogianni, Jan Mahlmann, Ralf Eberhardt

**Affiliations:** 1 Pneumology and Critical Care Medicine, Thoraxklinik, University Heidelberg, D-69126 Heidelberg, Germany; franziska.trudzinski@med.uni-heidelberg.de (F.C.T.);; 2 Translational Research Center (TLRC), German Centre for Lung Research (DZL), D-69126 Heidelberg, Germany; 3 Thoracic Surgery, Klinikum Rechts der Isar, Technical University Munich (TUM), D-81675 Munich, Germany; 4 Thoracic Surgery, Universitätsklinikum des Saarlandes, D-66421 Homburg/Saar, Germany; 5 Thoracic Surgery, Asklepios Klinik Barmbek, D-22307 Hamburg, Germany; 6 Pneumology and Critical Care Medicine, Asklepios-Klinik Barmbek, D-22307 Hamburg, Germany

We read with interest the study by Akil et al. comparing nonintubated versus intubated lung volume reduction surgery (LVRS) in patients with end-stage emphysema and hypercapnia [1]. The authors report on 36 patients who underwent nonintubated LVRS and 56 patients who received intubated LVRS, all with low-flow veno-venous extracorporeal lung support (low-flow VV ECLS), at Ibbenbüren General Hospital between April 2019 and February 2021. The study aimed to examine perioperative outcomes, including complication rates and postoperative recovery for both groups, but it fails to provide a compelling rationale for the overall indication of the chosen surgical approach.

The fact that a total of 92 patients underwent LVRS with ECMO support at a single institution in less than two years raises significant concerns regarding the chosen level of invasiveness at this center. Furthermore, the indication for LVRS is not clear to us on the basis of the values given, especially as patients with a 6 min walk distance of 450 m and an FEV1 of 68% predicted were considered for the procedure.

Nonintubated video-assisted thorascopic surgery (NI-VATS) has been demonstrated to be a safe and less invasive method for LVR. The absence of mechanical ventilation has the potential to protect fragile emphysemateous lung tissue from damage and to reduce postoperative air leaks and complications [2]. Whether this should be performed under ECMO is another question. Irrespective of whether the LVRS is intubated or nonintubated, the approach described by Akil et al. seems to be not only overly invasive but also questionable from a methodological point of view, especially since ECMO-assisted LVRS seems to be the standard procedure at this institution. The overall high number of LVRS procedures compared to other German centers is surprising and raises the question as to why less invasive alternatives, such as bronchoscopic lung volume reduction (BLVR) with valves, were apparently not considered in this risk group. This omission is particularly worrying as the indication for LVRS was made in an interdisciplinary panel where a more balanced assessment of therapeutic options would have been expected. Furthermore, there is a lack of data regarding the 3-month follow-up to demonstrate the efficacy of the procedure within this cohort to justify this invasive therapy. Well-documented data from the German Emphysema Registry strongly support the use of BLVR, especially hypercapnic patients [3]. Additionally, details regarding missing values and those lost to follow-up are absent, which is typically addressed in retrospective analyses.

Low-flow ECMO has been introduced as a promising technique for the treatment of hypercapnic respiratory failure [4], but its clear benefit in use has yet to be demonstrated. Both the ECLAIR and VENT-AVOID studies highlight the challenges associated with the use of low-flow VV ECLS or extracorporeal CO_2_ removal (ECCO2R) in patients with exacerbations of chronic obstructive pulmonary disease or hypercapnic respiratory failure. The ECLAIR study found that while ECCO2R avoided intubation in 56% of cases, it also resulted in significant complications in 44% of patients, including major bleeding in 36%. Despite a shorter duration of invasive mechanical ventilation in the ECCO2R group, there were no significant differences in hospital length of stay or mortality rates compared to standard care [5]. Similarly, the VENT-AVOID study demonstrated that ECCO2R did not significantly increase the number of ventilator-free days (VFD-5) compared to standard care, and it reported higher all-cause in-hospital mortality in the ECCO2R group within the NIV stratum (22% vs. 0%) [6]. These findings collectively indicate that there is currently no data-driven rationale to support the use of ECCO2R in lung volume reduction surgery (LVRS) or other contexts, as the lack of demonstrated superiority over conventional mechanical ventilation and the associated complications raise concerns about its safety and efficacy in this patient population.

Further research is needed to establish clear benefits before ECCO2R can be recommended as a standard treatment option. In light of these findings, we are surprised that the specific complications associated with ECCO2R, such as bleeding complications, thrombosis, and pulmonary embolism [7], are not mentioned. Given the significant rates of adverse events observed in the ECLAIR study, which had already been published at the time of publication of this paper, it is crucial to address these serious risks when considering the use of ECCO2R in LVRS in mild to moderate hypercapnia (mean preoperative PCO2 of 48.1 and 51.1 mmHg in both groups, respectively) [1]. Ignoring these potential complications could mislead clinicians and patients about the true safety profile of these interventions.

We acknowledge that this paper has been available since May 2023. However, we have only recently become aware of it, and we feel it is important to address the claims made in the publication. It is also conceivable that practices at this institution may have changed since this study was conducted. In summary, we consider the approach taken by this institution to be highly questionable and advocate for a more critical discussion of the role of ECMO-supported LVRS in patients with mild to moderate hypercapnic emphysema, especially with regard to established minimally invasive alternatives.
